# Carcinosarcoma of the ovary

**DOI:** 10.1038/sj.bjc.6600770

**Published:** 2003-03-04

**Authors:** M A Harris, L M Delap, P S Sengupta, P M Wilkinson, R S Welch, R Swindell, J H Shanks, G Wilson, R J Slade, K Reynolds, G C Jayson

**Affiliations:** 1 Department of Clinical Oncology, Christie Hospital NHS Trust, Wilmslow Road, Withington, Manchester M20 4BX, UK; 2 University of Manchester Medical School, Stopford Building, Oxford Road, Manchester, UK; 3 Department of Gynaecological Oncology, St Mary's Hospital, Oxford Road, Manchester, UK; 4 Medical Statistics, Christie Hospital NHS Trust, Wilmslow Road, Withington, Manchester M20 4BX, UK; 5 Department of Histopathology, Christie Hospital NHS Trust, Wilmslow Road, Withington, Manchester M20 4BX, UK; 6 Department of Histopathology, St Mary's Hospital, Oxford Road, Manchester, UK; 7 Department of Gynaecological Oncology Surgery, Christie Hospital, Wilmslow Road, Withington, Manchester, UK; 8 Department of Medical Oncology, Christie Hospital NHS Trust, Wilmslow Road, Withington, Manchester M20 4BX, UK

**Keywords:** ovarian cancer, carcinosarcoma, pathology, chemotherapy

## Abstract

We report our experience in the management of patients with carcinosarcoma of the ovary, a rare but aggressive variant of ovarian cancer. Forty patients were treated at a single centre, which is the largest reported series. The median age at diagnosis was 65 years (range 45–86) and the median Karnofsky performance (KP) status was 70. Thirty-two patients (80%) presented with FIGO stage III or IV disease. Twenty-four had heterologous and 14 homologous carcinosarcoma on review of histopathology, but there was no significant difference in survival between these groups (*P*=0.28). Twenty-seven of the 40 patients had bulk residual disease present after surgery and this was associated with a worse prognosis (*P*=0.045). Chemotherapy was given to 32 patients (80%) of whom 26 (81%) received platinum-based regimens. Of these 32 patients, three (9.4%) achieved a complete response (CR), 10 (31%) a partial response (PR), five (16%) had stable disease, 10 (31%) had progressive disease and four were not assessable. Of the 19 patients who had a CR, PR or stable disease after chemotherapy or were unevaluable (stage Ic), the median survival was 29.6 months. Currently, seven patients are still alive although one has cancer. The overall censored median survival was 8.7 months after a median follow-up of 34 months, and the 1- and 5-year survival were 40 and 7.5%, respectively.

Carcinosarcomas are rare tumours and pathologically consist of a mixture of malignant epithelial and malignant mesenchymal components. They can occur throughout the female genital tract but are found most commonly in the uterus (Newlands and Paradinas, 1995). Ovarian carcinosarcomas are very rare and account for only <1–2% of all malignant ovarian tumours (Iakovleva and Shendereva, 1978; Chang *et al*, 1995; Melilli *et al*, 1999). Consequently, their behaviour and management is controversial as most centres only treat small numbers over many years. A previous study from our centre (Prendiville *et al*, 1994) reported on 20 patients from the 1980s who were mainly treated with surgery and nonplatinum-based chemotherapy.

Macroscopically the tumours are solid and/or cystic, fleshy and haemorrhagic and frequently spread beyond the ovary and over peritoneal surfaces by the time of surgery. The malignant mesenchymal component of the tumour is described as heterologous or homologous. Heterologous elements are tissues not normally found in the ovary, for example, cartilage, bone, smooth or striated muscle and adipose tissue. If the sarcomatous part contains elements that resemble endometrial stromal sarcoma, fibrosarcoma or leiomyosarcoma (‘homologous’ to the Mullerian duct system), then it is described as homologous.

The stage classification used for carcinosarcoma of the ovary is the same FIGO system that is applied to the other ovarian adenocarcinomas. In comparison with the more common ovarian carcinomas, the response to therapy and overall survival are poor with median survivals of 4–14 months (Hanjani *et al*, 1983; Terada *et al*, 1989; Prendiville *et al*, 1994; Chang *et al*, 1995), although this may be confounded to some extent by the higher FIGO stage (Hanjani *et al*, 1983; Morrow *et al*, 1984). We report a retrospective review of the demography, management and outcome of 40 patients treated for carcinosarcoma at this institute over the last 10 years and compare this to the published literature.

## MATERIALS AND METHODS

Between January 1991 and January 2001, 47 patients with the diagnosis of carcinosarcoma of the ovary were identified through medical records, the ovarian cancer database or by the department of histopathology. Seven of these patients were excluded from analysis because upon review it was unclear if their tumour had arisen in the ovary or elsewhere in the gynaecological tract. All patients had their tumour tissue reviewed by a specialist oncological histopathologist, and in all cases the stage of disease was calculated from the operative findings at diagnosis, a staging CT scan of the abdomen and pelvis (with and without contrast) and a chest radiograph.

The case notes were reviewed and information regarding patient demography, presenting symptoms and signs, FIGO stage, histology, subsequent management and response to treatment were recorded and transferred to a database. All intervals were calculated from the date of operation. Survival statistics were calculated by a statistician who generated Kaplan–Meier curves.

## RESULTS

### Patients

The age at operation ranged from 45- to 86 years with a median age of 65, which is similar to previous studies (Prendiville *et al*, 1994; Chang *et al*, 1995). The Karnofsky performance (KP) status ranged from 30 to 90 with a median of 70. The FIGO stage at first chemotherapy is shown in Table 1

**Table 1:** Patient characteristics

Median age (range)	*65 (45–86)*
Median KP (range)	70 (30–90)
FIGO stage I	1
FIGO stage II	6
FIGO stage III	17
FIGO stage IV	15
Unknown stage	1
No residual disease post surgery	4
Microscopic residual disease (<2 cm)	9
Bulk residual disease (>2 cm)	27
Homologous tumour	14
Heterologous tumour	24
Unknown type of tumour	2

and although patients presented in all four stages, 80% (32 patients) presented with advanced FIGO stage III or IV disease. In one patient, the initial stage was unknown because she was only referred on relapse. Twenty-seven patients had bulk residual disease (>2 cm) left post initial surgery, nine had microscopic residual disease (<2 cm) and four had no residual disease detectable. The survival for patients with no bulk residual disease post surgery was significantly better than those with bulk residual disease (*P*=0.045) and the survival curves are shown in Figure 1

**Figure 1:**
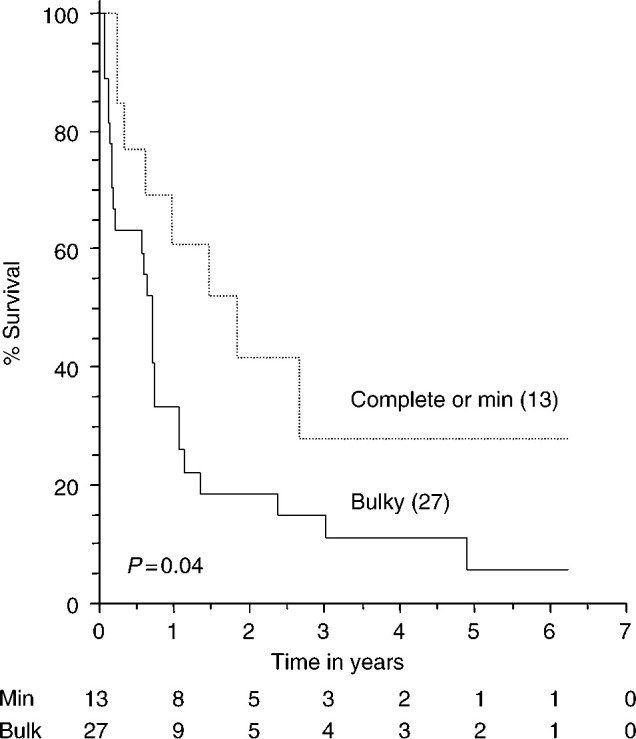
Relation between bulk residual disease and survival. The number of patients at risk are shown below the *x*-axis. The dotted line represents patients with complete cytoreduction and those with minimal residual disease. The solid line represents patients with bulky residual disease.

.

### Histopathology

Twenty-four of the patients were found to have heterologous elements and 14 had homologous carcinosarcoma on review of the histopathology. In two patients, despite review, it was impossible to identify which was present. The median survival for the patients with heterologous tumours was 8.5 months and for homologous tumours it was 8.9 months. The 1-year survival was 42% in the former group and 50% in the latter group. There was no significant difference in survival between the groups (*P*=0.28).

### Treatment

Chemotherapy was administered to 32 of the 40 cases (80%) and the remaining eight cases were either deemed too unwell or died before it could be given. Of the 32 patients who received chemotherapy, 26 (81%) cases received platinum-based regimens, mainly using carboplatin either alone or with ifosfamide (see Table 2

**Table 2:** Chemotherapy regimens

*Platinum containing chemotherapy regimens*	
Carboplatin and ifosfamide (Carbo-I)	9
Carboplatin (Carbo)	9
Cyclophosphamide, adriamycin and cisplatin (CAP)	3
Carboplatin and cyclophosphamide (Carbo-C)	2
Epirubicin, carboplatin and 5FU (E-Carbo-F)	1
Epirubicin, cisplatin and 5FU (ECF)	1
Taxol and Carboplatin (T-Carbo)	1
	
*Other chemotherapy regimens*	
Doxorubicin (A)	2
Doxorubicin and cyclophosphamide (AC)	1
Cyclophosphamide (IV)	1
Cyclophosphamide (oral)	1
Melphalan	1
*Total*	*32*

Carbo-I=carboplatin AUC of 5 and ifosfamide 3 g m^−2^ D1-3; Carbo=carboplatin AUC of 6; CAP=cyclophosphamide 750 mg m^−2^; doxorubicin 50 mg m^−2^ and cisplatin 50 mg m^−2^; Carbo-C=carboplatin AUC of 5 and cyclophosphamide 600 mg m^−2^; E-Carbo-F=epirubicin 50 mg m^−2^, carboplatin AUC of 5 and 5FU 1.2 m^−2^, ECF=epirubicin 50 mg m^−2^, cisplatin 70 mg m^−2^ and 5FU 200 mg m^−2^/day via a pump for 4 weeks repeated monthly; T-Carbo=taxol 175 mg m^−2^ and carboplatin AUC of 5; A=doxorubicin 75 mg m^−2^, AC=doxorubicin 60 mg m^−2^ and cyclophosphamide 600 mg m^−2^; cyclophosphamide=600 mg m^−2^ (IV) once every 3 weeks and 50 mg TDS for 14 days monthly; melphalan=5 mg for 5 days six times weekly. All chemotherapy were given once every 3 weeks unless stated.

). The dose of carboplatin given was calculated using an area under the curve of 6 when used as a single agent and 5 when used in combination, and ifosfamide was given at a dose of 3 g m^−2^ on days 1–3. Both chemotherapy regimens were given once every 3 weeks. Only one patient received a taxane because they were initially thought to have the more common type of ovarian adenocarcinoma. Of the 32 patients who received at least one cycle of chemotherapy, three out of 32 (9.4%) achieved a complete clinical and radiological response, 10 out of 32 (31%) achieved a partial response while in five out of 32 (16%) the disease remained stable and in ten out of 32 (31%) the disease progressed. Four of the 32 patients had disease that was not assessable as three died after the first cycle and one patient had FIGO stage Ic disease and had no radiologically detectable residual disease. Interestingly, one patient with stage III disease who received only one cycle of chemotherapy because of intercurrent illness appeared to have achieved a partial response on subsequent CT scans and is still alive 14 months later. For the six patients who received nonplatinum-based chemotherapy, two patients achieved a PR, three had progressive disease and one died after the first cycle and so was unassessable.

### Progression-free survival and the treatment of recurrent disease

Of the 19 patients who achieved either a complete response (CR) or partial response (PR) or static disease (including the patient with stage Ic), the median survival was 29.6 months and the time to recurrence (from date of operation) was 22.6 months. One patient was treated initially with pelvic radiotherapy and received chemotherapy later in the course of her disease. Four patients received radiotherapy for symptomatic residual disease (two for first recurrence and two for second recurrence) without evidence of response. Six patients underwent a further laparotomy after chemotherapy. Three of these patients had disease that was initially inoperable, but had a good response to chemotherapy and underwent definitive surgery that included a total hysterectomy, bilateral salpingo-oophorectomy and omentectomy. Of the other three, one underwent surgery for progressive disease immediately post chemotherapy, one at relapse several months later and the final patient had been treated elsewhere initially with oral chemotherapy, and had relapsed and undergone repeat laparo-tomy prior to referral to our centre. All three of these patients had initially had adequate debulking surgery.

### Overall survival

**Figure 2:**
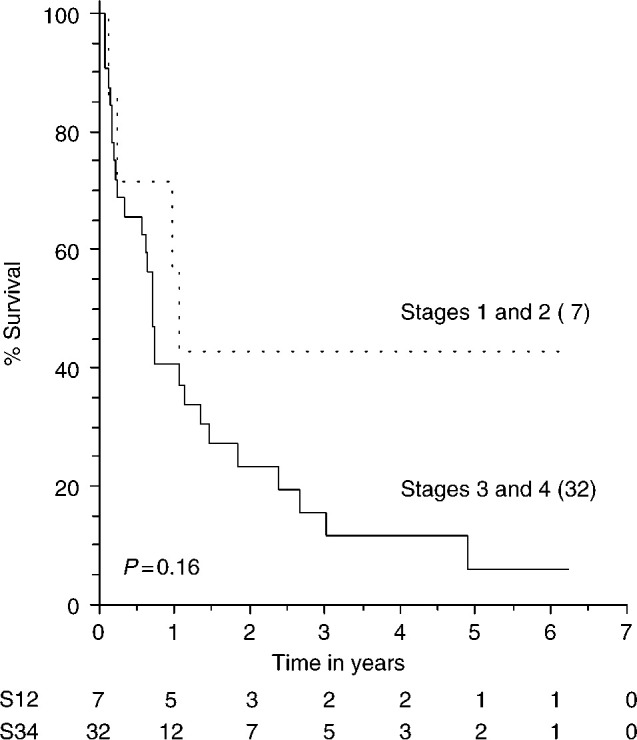
Relation between stage and survival. Survival was classified according to stages 1/2 and 3/4. The number of patients at risk are shown below the *x*-axis. The dotted line represents patients with stage 1 or 2 disease. The solid line represents patients with stages 3 and 4 disease. The stage in one patient could not be determined.

Currently seven patients are still alive, one with cancer, and there have been two intercurrent deaths. The other 31 patients have died of their disease. The overall censored median survival was 8.7 months and the median follow-up period from diagnosis was 34 months. The stage-related survival curves are shown in Figure 2

. There was no significant difference found between the survival curves for stages I and II *vs* stages III and IV (*P*=0.16).

## DISCUSSION

This is the largest reported series of carcinosarcoma of the ovary. The 40 cases made up 1.3% of the cases of malignant ovarian carcinoma in this institute over a 10-year period. The median age and stage at operation were similar to previous studies (Chang *et al*, 1995; Terada *et al*, 1989; Sreenan and Hart, 1995). It has been suggested that heterologous tumours may be more advanced and have a worse prognosis (Calame and Schaberg, 1989), but this was not confirmed by our results.

It is well known in ovarian carcinoma that bulk residual disease is associated with a worse survival, but this has not been shown statistically in ovarian carcinosarcoma before. Our data show a statistically significant survival benefit for women who have optimum cytoreduction after surgery.

The response rate of 41% in patients treated with chemotherapy compares favourably with the published data of 35–47% (Prendiville *et al*, 1994; Chang *et al*, 1995). The response rate in the 26 patients who received platinum-based chemotherapy was 42% and for other chemotherapy regimens it was 33%. Many different chemotherapy regimens were used reflecting the uncertainty of treatment for this malignancy. It was noteworthy that of the nine patients who were initially planned to receive the more toxic sarcoma-type chemotherapy with ifosfamide and carboplatin, six of them only tolerated one cycle with ifosfamide, and then either received no further chemotherapy or were treated with carboplatin alone. This reflects the poor general condition (median KP was 70) of these women. It was also interesting that responses to second- and third-line chemotherapy were observed particularly with a single-agent carboplatin.

The role of therapeutic radiotherapy is not established. In this series radiotherapy was given to only five patients – all for different indications and with no evidence of CRs. Three of these patients died within 6 months of radiotherapy and the other two relapsed less than a year post-treatment.

The overall median survival of 8.7 months is still poor as previously reported. The 1-year survival was 40% and the 5-year survival was 7.5% as compared to approximately 15–40% 5-year survival in epithelial ovarian carcinoma. As can be seen from the survival curves (Figure 1 and Figure 2), many patients die within the first few months of surgery and do not complete a course of chemotherapy, but there are a substantial subgroup of patients who respond to chemotherapy and appear to have a much better survival. Therefore, all patients with this diagnosis should be referred to a specialist centre for treatment, ideally within a defined protocol, although the optimal chemotherapy has yet to be fully identified. Clinical trials for carcinosarcoma of the ovary would be beneficial but may have difficulty accruing because of the rarity of the disease.
